# Loneliness before and after COVID-19: Sense of Coherence and Hope as Coping Mechanisms

**DOI:** 10.3390/ijerph20105840

**Published:** 2023-05-17

**Authors:** Michal Einav, Malka Margalit

**Affiliations:** 1 Behavioral Sciences Department, Peres Academic Center, Rechovot 7610202, Israel; einav.michal@gmail.com; 2 Behavioral Sciences Department, The Academic College of Tel-Aviv-Yaffa, Tel-Aviv 6818218, Israel; 3 School of Education, Tel Aviv University, Tel-Aviv 6997505, Israel

**Keywords:** loneliness, hope, sense of coherence, COVID-19, coping resources

## Abstract

The COVID-19 pandemic posed a major threat to public health, with long-lasting consequences for the daily habits and practices of people around the world. The combination of hazardous health conditions and extensive changes to people’s daily routines due to lockdowns, social restrictions, and employment uncertainty have led to mental health challenges, reduced levels of subjective wellbeing, and increased maladaptive behaviors and emotional distress. Nevertheless, some studies have reported increased adaptive functioning and resilience after the pandemic, suggesting a more complex pattern of effects. The goals of the current study were to explore the role of two coping variables, sense of coherence and hope, in people’s emotional wellbeing and adaptation in dealing with loneliness before and after such a stressful period. In a cross-sectional study, 974 Israeli participants (sample 1: 540 participants before the pandemic; sample 2: 434 participants after the pandemic restrictions) answered online questionnaires about their loneliness, hope and sense of coherence levels before and after the pandemic. While the two groups did not differ in their levels of hope, the participants in the group before COVID-19 reported lower levels of loneliness and sense of coherence. However, the results also indicated that although the COVID-19 pandemic was related to increased levels of loneliness, the participants’ sense of coherence mediated this increase and their levels of hope moderated it. The theoretical contribution of these findings is discussed, as well as interventional implications and future directions.

## 1. Introduction

Coping mechanisms are essential tools for individuals to deal with the stresses and challenges of life, specifically during times of hardship such as a global pandemic [1,2]. The COVID-19 pandemic has brought about unprecedented levels of uncertainty, fear, and anxiety throughout the world [3,4,5,6], emphasizing the critical role of effective coping strategies for promoting wellbeing and mental health. Past research has highlighted the critical role of various coping mechanisms in reducing stress and anxiety, providing a sense of control and stability during a time of uncertainty, helping manage anxious thoughts and negative emotions, promoting positive thinking and behavior, and encouraging social connections [7,8,9]. Focusing on coping mechanisms and promoting resilience has become particularly important, considering the emotional consequences of day-to-day changes and life threats during the COVID-19 pandemic.

The current study seeks to shed light on the coping mechanisms people used to mitigate the negative consequences of the COVID-19 pandemic. The results help us identify the factors that may predict resilience during times of major life-threatening distress.

## 2. Loneliness

Loneliness has been defined as the aversive emotional outcome experienced when a discrepancy exists between the interpersonal relationships people want to have, and those they currently experience [10]. Numerous studies have focused on the harmful consequences of prolonged loneliness, such as emotional distress, depression, and anxiety [11]. Left untended, loneliness may have serious consequences for people’s cognitive processes, emotions, behavior, and health.

The COVID-19 epidemic affected interpersonal and community interactions. Personal social contacts were dramatically reduced, to prevent the spread of the virus. The alternative remote forms of communication that were developed in place of face-to-face communication are regarded as more superficial and less intimate than personal social interactions [12].

Meta-analyses of the research surrounding the pandemic have examined the links between loneliness and the pandemic. However, surprisingly, they have yielded inconclusive results. Whereas some studies reported overall elevated levels of loneliness, as one might expect [12,13,14], others indicated that people’s levels of loneliness remained relatively stable [15,16].

In an attempt to identify the factors that account for the disparities in these findings, studies have generally focused on the risk factors that might account for these inconsistent reports. For example, researchers have established a positive association between low socioeconomic status and poor health and increased loneliness [17,18]. Similarly, mental health challenges such as depression, stress, anxiety, sleep disorders, and substance abuse were also associated with increased loneliness during the pandemic [18,19]. These studies concluded that individuals with various health and socioeconomic difficulties were at an increased risk of higher levels of loneliness during the pandemic. However, there is limited research on the role of the factors related to resilience, such as coping resources, in the links between the epidemic and loneliness.

The current study examines the significance of coping mechanisms using Antonovsky‘s salutogenic model. This health promotion paradigm emphasizes the factors that help people maintain their health and wellbeing rather than the factors that cause illness or distress [20]. Developed by Antonovsky, it is based on the belief that people have an innate drive to maintain their health and wellbeing, even in the face of adversity [21]. The term “salutogenic” comes from the Latin word “salus”, which means “health”, and “genesis”, which means “creation”. This dynamic construct proposes that people are not fully healthy or completely sick, but constantly move along a continuum between illness and health [20]. During periods of stress and uncertainty such as the pandemic, many people focused on the disease, its diagnosis, and impacts. Therefore, this emphasis on health promotion has a unique value. These attempts to identify how people can stay healthy and thrive, even during stressful times, can be empowering [21,22]. Therefore, rather than focusing solely on detecting or preventing adversity, the salutogenic model has been used extensively in a variety of settings, including healthcare [23], education [24], and workplace [25] health promotion programs, to help individuals and communities maintain and enhance their health and wellbeing. In line with this model’s tenets, the current study explores the role of two key resources: sense of coherence and hope.

### 2.1. Sense of Coherence (SOC)

Sense of coherence is considered a key factor in Antonovsky’s salutogenic approach that emphasizes the importance of promoting health [20,21]. SOC is defined as a person’s general orientation towards the world as comprehensible, manageable, and meaningful [20]. Thus, sense of coherence has three components. The first is people’s ability to understand their environment as ordered, consistent, and structured. Second, people expect their resources to enable them to meet their needs. Some of these resources may be under their own control, whereas others, such as professional support or the support of friends, may be available from other people. The third aspect refers to meaningfulness: the consideration of many aspects of their life as important and worthy of emotional commitment, engagement, and investment. Therefore, sense of coherence does not refer to a particular style of coping. Rather, it involves a broad repertoire of coping strategies from which people can choose the appropriate one in any given situation [21].

We posited that sense of coherence might be an important resource for coping with the pandemic because of COVID-19′s perception as a significant, unrelenting crisis. Earlier research has examined sense of coherence as a major factor in protecting people’s mental health. For example, studies established a relationship between lower levels of sense of coherence and higher levels of psychological distress [26]. In contrast, higher levels of sense of coherence were strongly associated with fewer symptoms of anxiety and depression [27]. In addition, sense of coherence buffered work-related burnout [28], a finding of particular significance, considering the increased vulnerability that medical teams faced due to exposure to the disease, their physical and emotional burdens, lack of resources, and exposure to death. This pattern of results, establishing the mediating role of sense of coherence in the relationship between pandemic experiences and psychological wellbeing, was also found in the general population, predicting mental health difficulties as well as future related anxiety [22,29]. In effect, major life changes during the pandemic disrupted people’s ability to consider future opportunities, a significant factor in wellbeing [30]. Therefore, we also examined the role of hopeful thinking about the future, assessed in people’s ability to identify their goals and plan for the future.

### 2.2. Hope

Hopeful thinking enables individuals to set goals, plan paths to achieving these goals, and gather the motivation and personal energy to follow them [31]. Hope theory emphasizes that it is a constant, structured, goal-oriented cognitive set. According to this premise, hopeful thinking incorporates two interrelated patterns of thinking: agentic thinking and pathways thinking. The first addresses the driving force in defining and achieving one’s goals, whereas the latter is focused on planning the paths to success and considering alternatives to possible barriers to achieving it [32]. Hence, researchers have proposed hope as a resilience factor, promoting people’s wellbeing and supporting their ability to cope effectively with stress [33,34].

Given the ongoing stress that the COVID-19 pandemic created, studies have investigated the role of hope as a protective factor during it. Recent studies have reported that hope is related to fundamental indicators of mental health such as satisfaction with life and a sense of meaningfulness [35], transcendent values and spiritual moorings [36], as well as resilience and behaviors to prevent stress [37]. One study of particular interest in this regard indicated that hope moderates the relationship between psychological distress and depression as well as between distress and insomnia. Thus, research has established the ability of hope to activate mechanisms that moderate psychological distress, especially in times of elevated fear and uncertainty [37,38,39,40].

### 2.3. The Current Study

The purpose of this study is to clarify the relationship between people’s reactions to the pandemic and their feelings of loneliness, and the role of hope and SOC in these relationships. Research has already demonstrated the role of SOC as a mediator in the relationship between various types of distress and outcomes [27,28,29,30] as well as the role of hope in moderating this relationship [41,42]. These studies presented SOC as a mediator with a direct path to the outcome. People who have higher levels of SOC are expected to be less lonely. Nevertheless, the early research on hope presented it as a mediator in several studies. Given that the anxiety and distress following COVID-19 caused many people to have difficulty thinking about and planning for their future, we hypothesize that experiencing medium and high levels of hope will activate SOC, leading people to feel less lonely. Thus, we expect people with medium and high levels of hope to have higher levels of SOC, which, in turn, will predict lower levels of loneliness [38]. Therefore, in line with the salutogenic approach [20], the current study has two main purposes: (1) to compare people’s levels of loneliness, SOC and hope before and after the pandemic and (2) to examine the mediating role of SOC and the moderating role of hope, as two key personal resources, in the relationship between the two periods and loneliness. We hypothesize that the availability of personal resources, specifically, sense of coherence and hope, will predict people’s levels of loneliness.

## 3. Method

### 3.1. Procedure

We utilized a cross-sectional repeated design consisting of two independent samples, one before and one after the pandemic’s restrictions and lockdown policies. The sample before the pandemic was collected between December 2017 and March 2018 as part of a larger study. The sample after the pandemic was collected during the end of January 2021, after the end of several periods of lockdowns, when life in Israel returned to normal. The college’s ethics committee approved these studies, and informed consent was obtained from all participants. Participation was voluntary and without compensation.

### 3.2. Participants

To test our hypothesis, we combined a sample of 974 Israeli participants from two samples: 540 participants before the pandemic and 434 participants after it. We used convenience sampling and sent the questionnaires to the participants via an electronic link on Qualtrics. Of those who participated before the pandemic, 59.4% (N = 321) were males, and after the pandemic, 54.4% (N = 236) were males. On average, the participants were 37.25 years old (SD = 11.86), and a large percentage (75.2%) had a university degree. The remaining participants (24.8%) had a high school diploma or technical education. Comparisons between the periods did not reveal significant differences in the proportions of the respondents by age, gender, or education.

### 3.3. Measures

We used several established measures to assess the respondents with regard to our variables.

***Loneliness.*** We used the Hebrew adaptation [43] of the loneliness scale [44] consisting of 11 statements that describe social and emotional loneliness (e.g., “I miss having a really close friend”). The respondents were asked to indicate the degree to which they agreed with each statement on a 5-point Likert scale ranging from 1 (not at all) to 5 (exactly like it). Cronbach’s α reliability for the scale was 0.83.

***Sense of Coherence*.** To assess sense of coherence [20,21] we used the short version of the self-report scale that taps three components: comprehensibility, manageability, and meaningfulness. The 10 statements were assessed on 7-point Likert-type scales. For example, the statement, “Until now your life has had: …” was rated on descriptors ranging from 1 (“no clear goals or purpose at all”) to 7 (“very clear goals and purpose”). Cronbach’s α reliability for the scale was 0.70.

***Hope.*** We used the Hebrew adaptation [45] of the State Hope Scale [31] consisting of six statements assessing hopeful thinking (e.g., “Even when others want to give up, I know I can find ways to solve the problem”). The respondents were asked to indicate the degree to which they agreed with each statement on a 6-point Likert scale ranging from 1 (never) to 6 (always). Cronbach’s α reliability for the scale was 0.87.

### 3.4. Data Analysis

**Figure 1:**
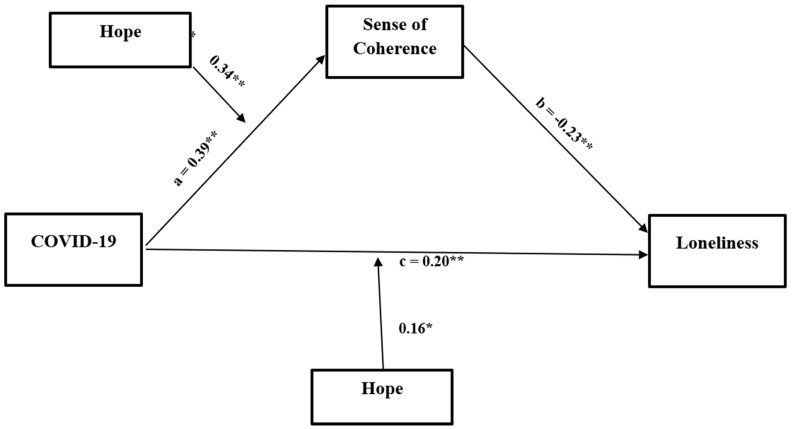
Direct and indirect effect of pandemic periods on loneliness through sense of coherence with three levels of hope moderating the path between periods and sense of coherence, and between periods and loneliness. a’= Low: 0.13*; Medium: 0.39**; High: 0.65**; c’ = Low: −0.08; Medium: 0.20**; High: 0.33**. Note: * *p* < 0.05; **p < 0.01.

As a preliminary analysis, we performed a MANOVA and calculated Pearson’s correlations. The hypothesized moderated mediation model (see Figure 1) was tested in a single model using a bootstrapping approach to assess the significance of the indirect effects at differing levels of the moderator [46]. The periods (before/after COVID-19) were the predictor variable, with sense of coherence as the mediator, and age and gender as covariates. The outcome variable was loneliness and hope was the moderator. Moderated mediation analyses tested the conditional indirect effect of the moderating variable (i.e., hope) on the relationship between the predictor (i.e., periods before/after the pandemic) and the outcome variable (i.e., loneliness) via a potential mediator (i.e., sense of coherence). The PROCESS macro, model 8, v 4.0 [47] in SPSS ver. 28 with bias-corrected 95% confidence intervals was used to test the significance of the indirect (i.e., mediated) effects moderated by hope, meaning the conditional indirect effects. This model tested the moderating effect on the predictor to mediator path (i.e., path a) and on the outcomes path. An index of moderated mediation was used to test the significance of the moderated mediation, meaning the difference in the indirect effects on three levels of hope [48]. Significant effects were supported by the absence of zero within the confidence intervals.

## 4. Results

### 4.1. Preliminary Analyses

In order to explore differences in the research variables during the two periods, we performed a one-way MANOVA with the periods (before/after the pandemic) as the independent variables, and loneliness, hope, and sense of coherence as the dependent variables. Age and gender were controlled. A main effect for the periods emerged F (3, 967) = 30.49, *p* = 0.001, partial η^2^ = 0.086. A univariate analysis yielded a main effect for loneliness (before COVID-19: M = 2.37, SD = 0.81, after COVID-19, M = 2.48 SD = 0.73, F (1, 973) = 5.57, *p* = 0.002, partial η^2^ = 0.006) and sense of coherence (before COVID-19: M = 4.41, SD = 0.76, after COVID-19, M = 4.80, SD = 0.82, F (1, 973) = 60.54, *p* = 0.001, partial η^2^ = 0.059), but not for hope.

Pearson’s correlations between the research measures were also calculated, revealing negative correlations between sense of coherence and loneliness (r = −0.27, *p* < 0.01), negative correlations between loneliness and hope (r = −0.32, *p* < 0.01), and positive correlations between hope and sense of coherence (r = 0.39, *p* < 0.01).

### 4.2. Moderated Mediation Analyses

Using the PROCESS macro model, the hypothesized moderated mediation model (model 8) was tested. In accordance with our model in Figure 1, there were significant relationships between the pandemic periods and loneliness (path c: B = 0.20, se = 0.05, t = 4.26, *p* = 0.00, CI = 0.1100; 0.2981). In addition, sense of coherence mediated these relationships in path A (the path of the periods to sense of coherence: B = 0.39, se = 0.05, t = 8.69, *p* = 0.00, CI = 0.2993; 4738) and path B (the path of sense of coherence to loneliness: B = −0.22, se = 0.03, t = −6.71, *p* = 0.00, CI = −2894; −1584). Hope moderated the effect of COVID-19 with sense of coherence (Interaction B = 0.34, se = 0.06, t = 5.61, *p* = 0.00, CI = 0.2197; 0.4560) and COVID-19 with loneliness (Interaction B = 0.16, se = 0.06, t = 2.52, *p* = 0.01, CI = 0.0352; 0.2844). The overall moderated mediation model was supported by the index of moderated mediation = −0.0756 (95% CI = −0.1146; 0.0415).

**Figure 2:**
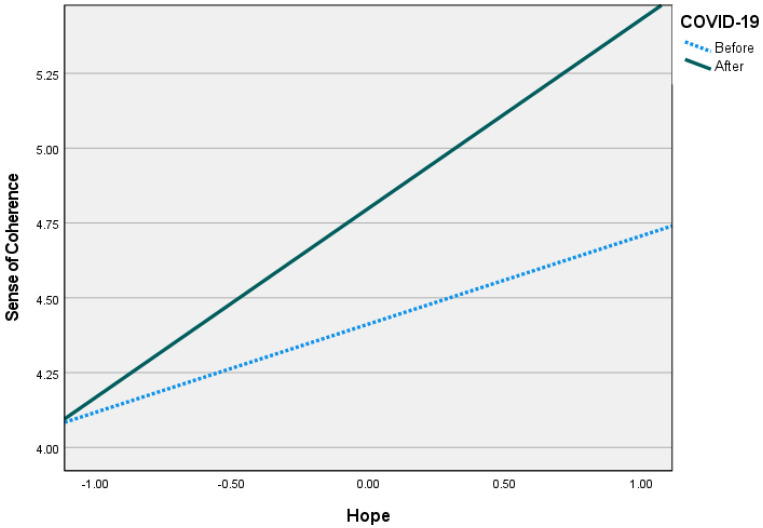
Hope as a moderator that differentiated sense of coherence levels during the periods.

**Figure 3:**
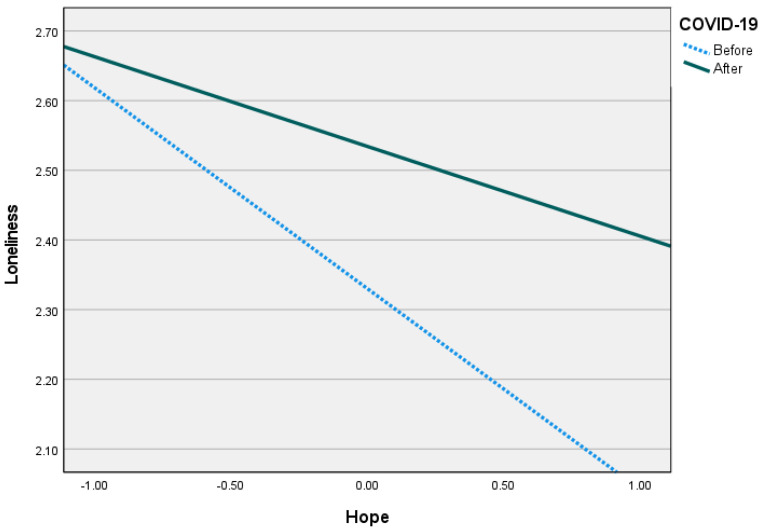
Hope as a moderator that differentiated loneliness levels during the periods.

As Figure 2 indicates, overall, there was a greater sense of coherence after the pandemic. However, individuals with higher levels of hope reported a stronger sense of coherence than those with lower levels of hope (Low hope: B = 0.13*, Medium hope: B = 0.39**, High hope: B = 0.65**). Figure 3 demonstrates that after COVID-19, people felt lonelier than before it. However, in both periods, individuals with medium to high levels of hope reported less loneliness than those who felt less hopeful (Low hope: B = −0.08, Medium hope: B = 0.20**, High hope: B = 0.33**). Thus, after the pandemic period, people tended to feel lonelier than before it. However, that feeling was mediated by their sense of coherence and moderated by their levels of hope. Therefore, while COVID-19 predicted higher levels of sense of coherence, those with more hope were less lonely than those who felt less hopeful.

## 5. Discussion

The COVID-19 pandemic brought about significant changes in the way individuals live their lives, including social distancing, lockdowns, and isolation. These changes have had a profound impact on people’s mental health, with many experiencing increased feelings of loneliness [12].

Consistent with past research [13,14], the results of the current study indicated that the COVID-19 pandemic was related to increased levels of loneliness. In other words, many participants reported feeling lonelier after the pandemic than before it. This finding supports previous studies and emphasizes the harmful social consequences of the pandemic, which forced individuals to endure prolonged periods of social distancing and isolation.

However, some studies also indicated that the link between the pandemic and loneliness varied among people [15,16]. In an attempt to explore these interpersonal variations, our findings revealed that SOC and hope mediated and moderated this link, respectively. Rather than focusing on issues related to the disease of COVID-19 and the distress it created, we utilized the salutogenic approach and explored the factors that empowered people to deal with the situation and promoted their health.

Within this construct, as a personal strength, SOC may enable people to understand and make sense of the world around them. Thus, when faced with a crisis, those who could rely on their sense of coherence were less paralyzed by stress. They were better able to deal with the adversity and be more resistant to loneliness. They were also able to continue the move towards maintaining and improving their mental health.

The results also indicated that hope moderated the links between the pandemic periods and sense of coherence and those between the pandemic periods and loneliness. Therefore, those individuals whose SOC reflected the impact of the distress were able to benefit from medium and high levels of hopeful thinking. They were able to activate their SOC and thus minimize their feelings of loneliness in the face of adversity. This finding further establishes the importance of hope as a fundamental personal activating strength that enables people to have a vision of the future even in the face of a challenging, immobilizing present. It is the basis of the ability to cope with, adjust to, and endure difficult times.

Together, these results emphasize the contribution of the salutogenic approach to promoting wellbeing. Both sense of coherence and hope can help people develop a sense of purpose and meaning in life, which can reduce feelings of loneliness. Studies have documented these links, emphasizing the valuable relationship between sense of coherence and the ability to find life meaningful [49,50] as well as the facilitating effect of the latter on loneliness [51,52]. Given these findings, individuals with a strong sense of coherence may be better able to make sense of the changes brought on by COVID-19 and adapt to new ways of connecting with others. Similarly, those who have hope for the future may be more likely to seek out social connections and find ways to maintain those connections during times of social distancing. Overall, having a strong sense of coherence and hope can be beneficial for coping with loneliness after COVID-19. Thus, from an empirical standpoint, interventions that strengthen these qualities, such as workshops on developing hopeful thinking, cognitive behavioral therapy, or mindfulness-based stress reduction techniques are positive steps for improving people’s wellbeing. For instance, studies have documented how intervention programs designed to improve people’s levels of hope [53,54] can help them deal with the stress and challenges of the COVID-19 pandemic. These interventions may target hope-based cognitive strategies for positive thinking, problem solving, and goal setting, providing people with the tools to manage the stress and uncertainty of the pandemic. Additionally, such interventions can help reduce negative emotions such as helplessness and apprehension, which may be exacerbated by the pandemic, by promoting positive emotions and outlooks during challenging times. Lastly, with regard to loneliness specifically, interventions to increase people’s levels of hope can help them build social support networks by strengthening their participatory communication and active listening skills, encouraging the inclusion of the feelings of others, and motivating participants to reach out to friends, family or colleagues.

Taking a broader point of view, the COVID-19 pandemic is an extreme example of an adverse situation affecting individuals and communities alike. Allocating resources to develop and strengthen people’s resilience will help them avoid the detrimental effects of crises and grow even stronger in the future. As our results suggested, the availability and accessibility of coping mechanisms are significant in dealing with adversity. They can help reduce the negative effects of stress, build resilience, develop problem-solving skills, foster social support, and minimize loneliness. By utilizing effective coping mechanisms, individuals can better manage the challenges they face and maintain their mental health and wellbeing.

## 6. Limitations and Future Directions

This study has several limitations. First, it was based on self-reported, correlational research, potentially raising concerns regarding causality and social desirability bias. In addition, our cross-sectional study used two convenience samples based on online questionnaires. The two samples were collected in an identical manner. We ensured the anonymity of the participants by using online questionnaires, but doing so limited the personal information available to us. Thus, future studies should validate the results by using observational methods, in-depth interviews, or a combination of research methods. In addition, we collected the second sample soon after people returned to their normal life. Future studies should investigate the long-term impacts of the pandemic and the role of the resilience factors we explored. Additional resilience factors, both intrapersonal ones (e.g., attachment style) and interpersonal ones (e.g., family and social support), may extend our understanding of people’s resilience and adjustment to adverse situations.

## Data Availability

The data presented in this study are available on request from the corresponding author. The data are not publicly available due to participants’ privacy concerns.
